# Effects of antibiotics ceftriaxone and levofloxacin on the growth of *Protophormia terraenovae* (Diptera: Calliphoridae)

**DOI:** 10.1007/s12024-024-00804-9

**Published:** 2024-03-26

**Authors:** Daniel Preußer, Thomas Fischer, Thomas Juretzek

**Affiliations:** 1 Chair of Ecology, Brandenburg University of Technology Cottbus-Senftenberg, Konrad-Wachsmann-Allee 6, 03046 Cottbus, Germany; 2 Central Analytical Laboratory, Brandenburg University of Technology Cottbus-Senftenberg, Konrad-Wachsmann-Allee 6, 03046 Cottbus, Germany; 3 Study Program Forensic Sciences and Engineering, Erich-Weinert-Str. 1, 03046 Cottbus, Germany; 4 Carl-Thiem-Klinikum Cottbus, Thiemstraße 111, 03048 Cottbus, Germany

**Keywords:** Antibiotics, Blowflies, Post-mortem interval, Entomotoxicology

## Abstract

*Protophormia terraenovae* is a colonizer of decomposing bodies and is known to cause pre-mortem myiasis as the female flies lay eggs in uncleaned wounds. In this study the effects of different concentrations of antibiotics levofloxacin and ceftriaxone on maggot development, weight, length, and mortality were examined. The maggot length and weight were significantly increased by therapeutical doses of levofloxacin and ceftriaxone. The maggot development time was significantly decreased in every levofloxacin treatment compared to the control. The time to start pupation was significantly increased in the control compared to the antibiotic treatments. Levofloxacin significantly increased the survivability of the maggots. Every levofloxacin treatment significantly improved the rearing conditions for the maggots. Reaching the third instar was delayed by 24 h in the control compared to the Levo 3.57 treatment. The Pupation in the control was delayed by an average of 48 h compared to the Levo 3.57 treatment. The significantly reduced development time of the maggots in the antibiotic treatments might lead to an overestimation of the post-mortem interval and therefore an incorrect time of death determination. The improved rearing conditions may be an indication of the potential of a combined application of antibiotics and maggot therapy.

## Introduction

*Protophormia terraenovae* (Robineau-Desvoidy, 1830) is a Calliphoridae species with a growing importance in medical and forensic entomology due to its necrophagous properties [1–7]. The species is known to cause pre-mortem myiasis and is a vector for bacterial based diseases [8, 9]. Recent studies have tested the ability of wound cleaning properties of maggot therapy with *Protophormia terraenovae* [10, 11]. The species is distributed in the Holarctic region and is commonly found in cold regions [10, 12]. Due to the importance of *Protophormia terraenovae* in forensic entomology, studies have been conducted to analyze the effects of temperature on the maggot development [1, 3, 12]. Additional studies have tested the effects of temperature and photoperiod on the adult flies [13]. A study on the effects of methylparaben on the maggot development revealed a prolonged development time, as well as a reduced size of the pupae and an increased pupae mortality [14]. Effects of antibiotics on the maggot development of *Protophormia terraenovae* haven’t been tested yet, however Preußer et al. (2021, 2023) examined the effects of ceftriaxone and levofloxacin on maggot development of *Lucilia sericata* (Meigen, 1826) and *Calliphora vomitoria* (Linnaeus, 1758) [15, 16]. The effects differed significantly depending on the examined species as levofloxacin decreased the maggot size, delayed the time to start pupation and increased the survivability of *Calliphora vomitoria*. In contrast, the antibiotics decreased the survivability of *Lucilia sericata* and did not significantly alter the maggot length and weight [16].

To analyze the effects of antibiotics levofloxacin and ceftriaxone on maggot development, survivability, length, and weight, tests were conducted with *Protophormia terraenovae* maggots. On the one hand, symbiotic bacteria have an important role in the digestion and immune system of the host [17, 18], on the other hand, bacterial pathogens can delay the insect development or kill them [19–21]. Antibiotics might therefore impair the rearing conditions by killing beneficial intestinal flora or improving the rearing condition by killing pathogenic bacteria. Antibiotics are a commonly used medication class with 70 billion prescriptions in 2011 [22]. A study conducted by Vaughn et al. revealed an increase in the global antibiotic use since 2000 by 46% [23]. The half-life of levofloxacin and ceftriaxone is between 7 and 8 h [24]. Renal insufficiency increases the half-life of levofloxacin up to 24–40 h [25]. The half-life of ceftriaxone increases up to 16–24 h among patients over the age of 75 years [26]. The chemical structure of both antibiotics remains unaltered after the administration. The secretion of levofloxacin takes place in the kidneys (85%). The secretion of ceftriaxone takes place in the liver (40–50%) and the kidneys (50–60%). As antibiotics may alter the rearing conditions for *Protophormia terraenovae*, we evaluated the hypothesis that therapeutical doses delay the maggot development, and cause therefore a wrong determination of the post-mortem interval. Three concentrations of each antibiotic and a mixed treatment were used to analyze the effects of levofloxacin and ceftriaxone on the development of *Protophormia terraenovae*, the results were compared using log-rank tests and two-way ANOVA.

## Materials and methods

### Experimental setup

A detailed description of the experimental design as well as a description and the results of the preliminary tests to examine the duration of the bactericidal effects of antibiotics in non-living tissue are described by Preußer et al. [15]. In brief, two experimental factors were examined. Factor A describes whether and what type of antibiotic treatment (none, ceftriaxone, levofloxacin, or mixture) was added to the nutritional basis and factor B describes the antibiotic concentration. The antibiotics were dissolved in water and mixed into the meat with a hand blender. The meat in the control was also prepared with a hand blender as the process altered the consistency of the meat. The used amount of levofloxacin and ceftriaxone is described in Table 1, the concentrations were chosen based on the secretion inside a living human. Assuming a half-live of 8 h for both antibiotics, the concentration of levofloxacin would decrease from the initial effective concentration of 3.57 µg g^-1^ to 1.79 µg g^-1^ to 0.89 µg g^-1^ and the concentration of ceftriaxone would decrease from the initial concentration of 28.57 µg g^-1^ to 14.29 µg g^-1^ to 7.14 µg g^-1^. After 24 h, another application of antibiotics would be administered and a concentration of approximately 3.57 µg g^-1^ levofloxacin or 28.57 µg g^-1^ ceftriaxone would be obtained again, and the process would continue until the medication is stopped.

**Table 1:** Amounts added to samples and effective initial concentrations of ceftriaxone (Cef) and levofloxacin (Levo) representing antibiotic treatment 2, 10 and 18 h ago assuming a half life time of 8 h and a resorption delay of 2 h (from Preußer et al. 2021) [15]

Treatment	Factor A (Antibiotic)	Hours since last antibiotic treatment prior to death	Amount added to 25 g of minced meat [µg]	Factor B (effective initial concentration) [µg g^− 1^]
Control	Control		0	0
Cef2857	Cef	2	714	28.57
Cef1429		10	357	14.29
Cef0714		18	179	7.14
Levo357	Levo	2	89.3	3.57
Levo179		10	44.6	1.79
Levo089		18	22.3	0.89
Mix	mixed	2	714 (Cef) 89.3 (Levo)	28.57 (Cef) 3.57 (Levo)

### Main test procedure

The flies were identified according to Szpila (2020) [27] and species validation was conducted by Prof. Dr. Jens Amendt of the Institute of Forensic Medicine at Goethe-University Frankfurt (Germany). Petri dishes with meat and the described amount of antibiotics were prepared according to Preußer et al. and 30 freshly hatched and vital maggot were distributed on each sample [15]. The samples were placed in cylindrical boxes and kept in an incubator at a temperature of 25 ± 1 °C. Paper towels inside the boxes provided dry hiding places once the maggots reached the post-feeding stage. The maggots were examined at 24-hour intervals. The maggots were carefully cleaned from the adhering substrate and the length, weight, development stage and number of living maggots were measured. Every measurement was conducted on living maggots to examine the development from the hatching to the pupation instead of using separated populations. Additionally, depending on the preservation and the killing method of the maggots, the maggot length can be altered after death [28, 29]. The examination was stopped when the maggots started pupating. 6 replicates of the main test procedure were conducted to determine the effects on 180 maggots per treatment, resulting in 1440 maggots overall.

### Data analysis

All statistical evaluations were conducted with the R software and a significance level of *p* = 0.05 was applied for all comparisons. Robust two-way ANOVA was used to evaluate the interaction between the two factors “duration” and “treatment.” of the maggot weight and length The mcp2a function included in the WRS2 package was used to conduct the post hoc tests. Log-rank tests along with Kaplan-Meier curves were used to compare the pupation and survival of the maggots [30, 31].

## Results

### Maggot weight

**Fig. 1:**
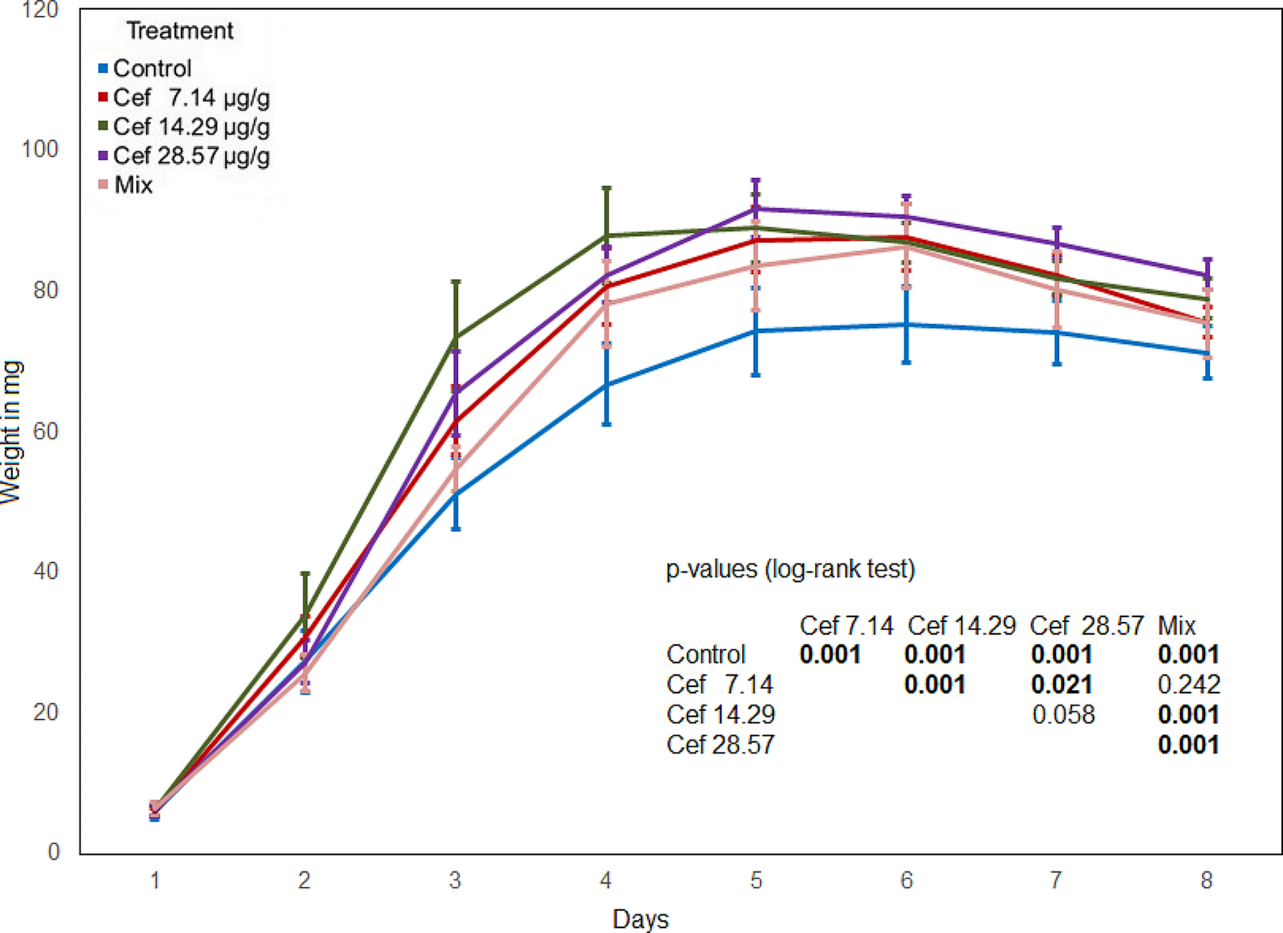
Maggot weight (ceftriaxone), bold numbers indicate significant differences

**Fig. 2:**
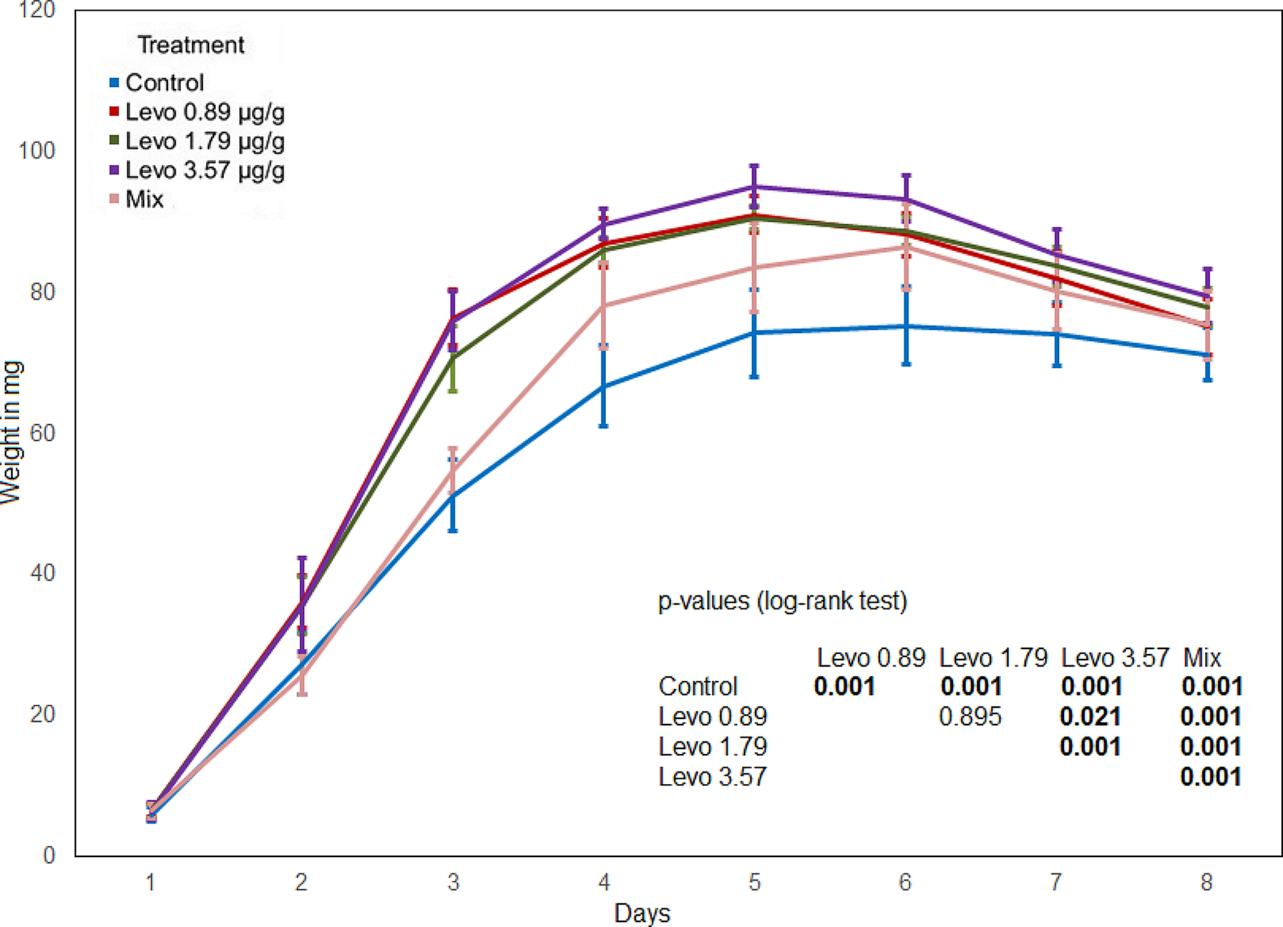
Maggot weight (levofloxacin), bold numbers indicate significant differences

The maggot weight in the control was significantly lower compared to every antibiotic treatment. The maggot weight in the mixed treatment was significantly lower compared to all levofloxacin treatments and the Cef 14.29 and Cef 28.57 treatments (Figs. 1 and 2). The maggot weight in the Levo 3.57 treatment was significantly higher compared to the Levo 0.89 and Levo 1.79 treatments. The comparison of the Levo 0.89 and Levo 1.79 treatments shows no significant difference (Fig. 2). From the first to the fourth day, the highest weight was determined in the Cef 14.29 treatment, and from the fifth to the eight day in the Cef 28.57 treatment, no significant difference was found between the two treatments. The maggot weight in the Cef 7.14 treatment was significantly lower compared to the Cef 14.29 and Cef 28.57 treatments.

### Maggot length

**Fig. 3:**
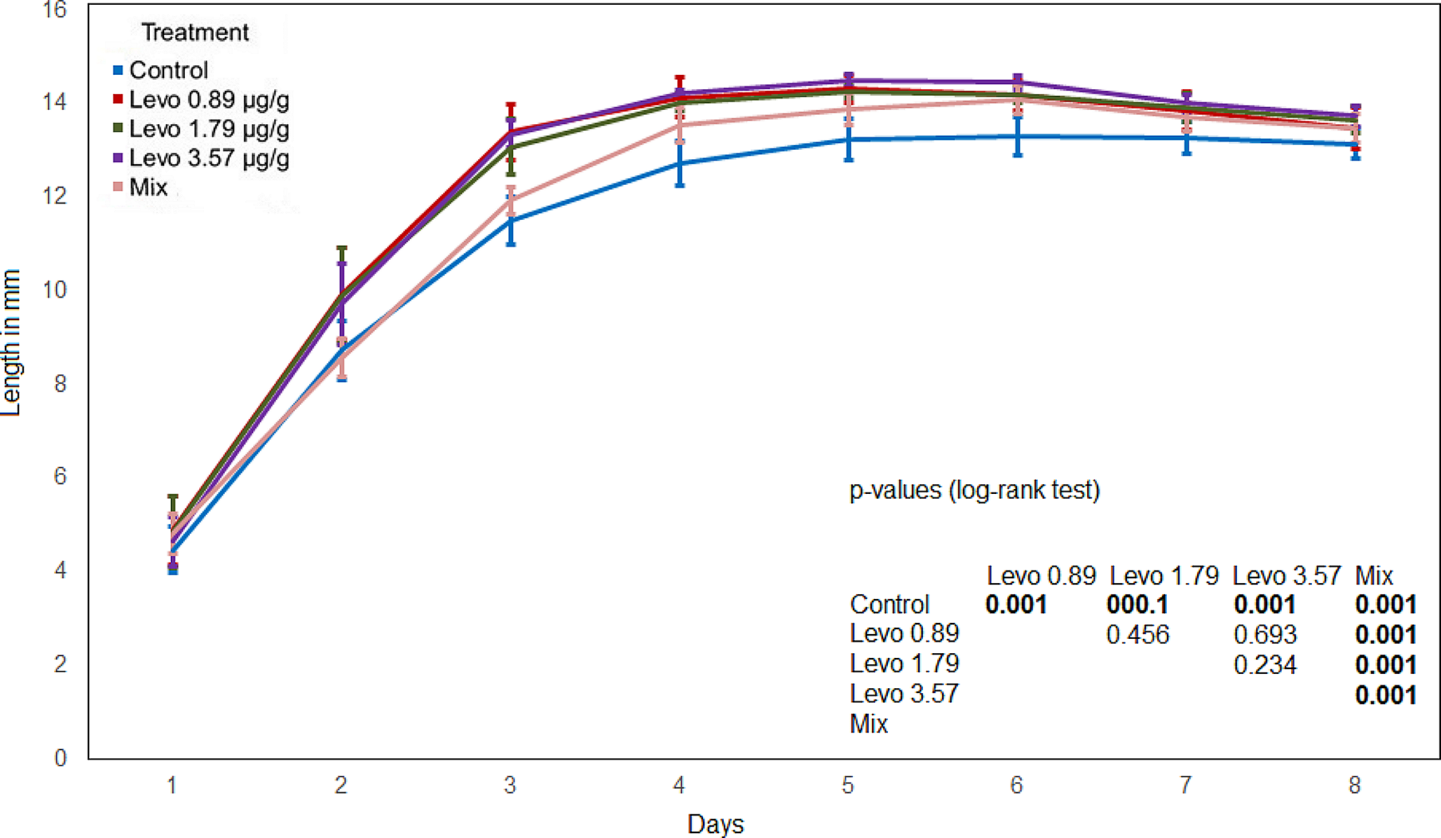
Maggot length (levofloxacin), bold numbers indicate significant differences

**Fig. 4:**
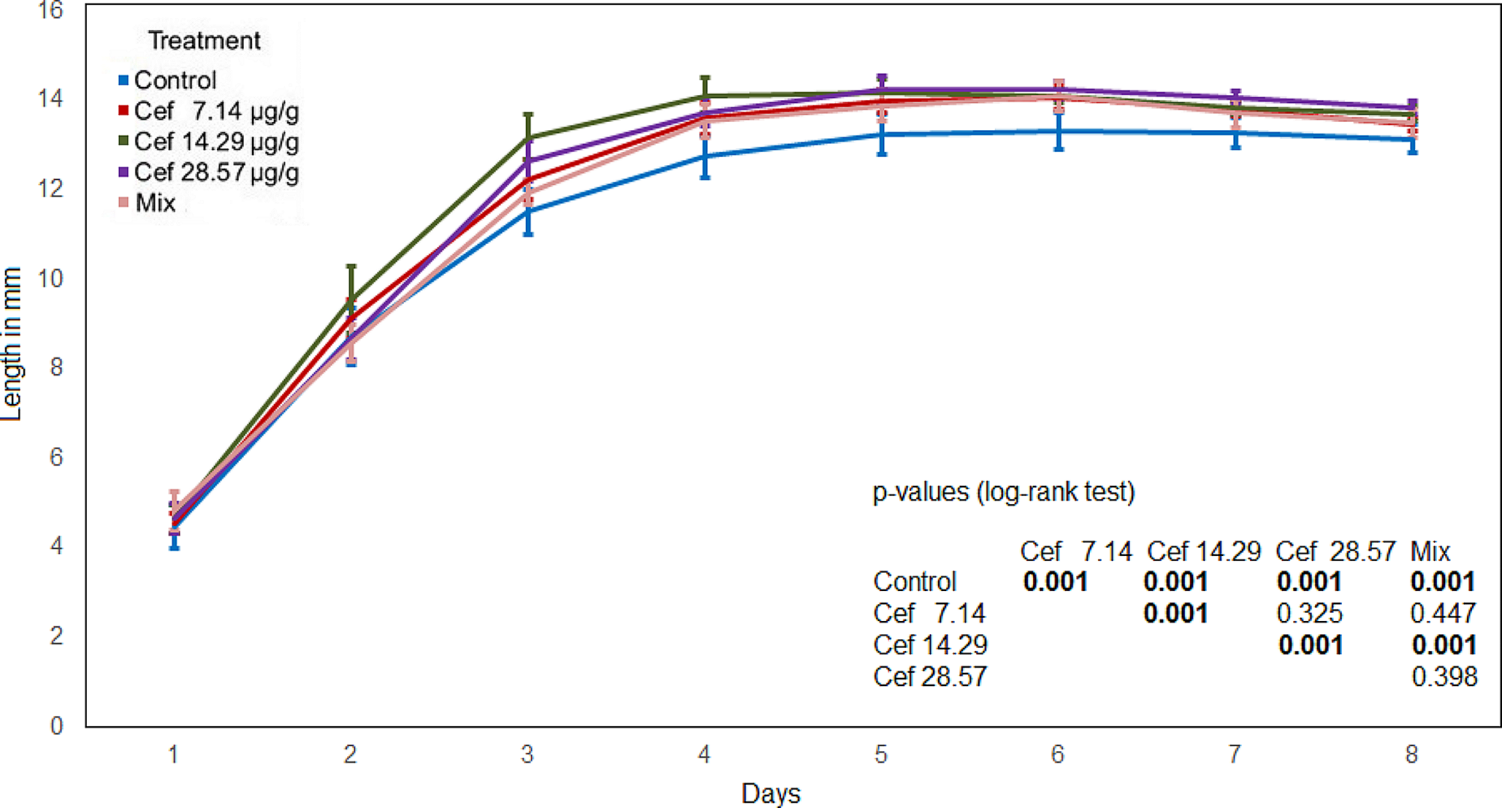
Maggot length (ceftriaxone), bold numbers indicate significant differences

A significant correlation between the length and the weight of the maggots was determined (Spearman’s rho > 0.95*** for both antibiotics). The maggot length in the control was significantly lower compared to every other treatment. The maggot length in the mixed treatment was significantly lower compared the levofloxacin treatments and the Cef 14.29 and Cef 28.57 treatments. The comparison of the levofloxacin treatments shows no significant differences (Fig. 3). The maggot length in the Cef 7.14 treatment was significantly lower compared to the Cef 14.29 and Cef 28.57 treatments. The comparison of the Cef 14.29 and Cef 28.57 treatments shows no significant difference (Fig. 4).

### Development stages

**Fig. 5:**
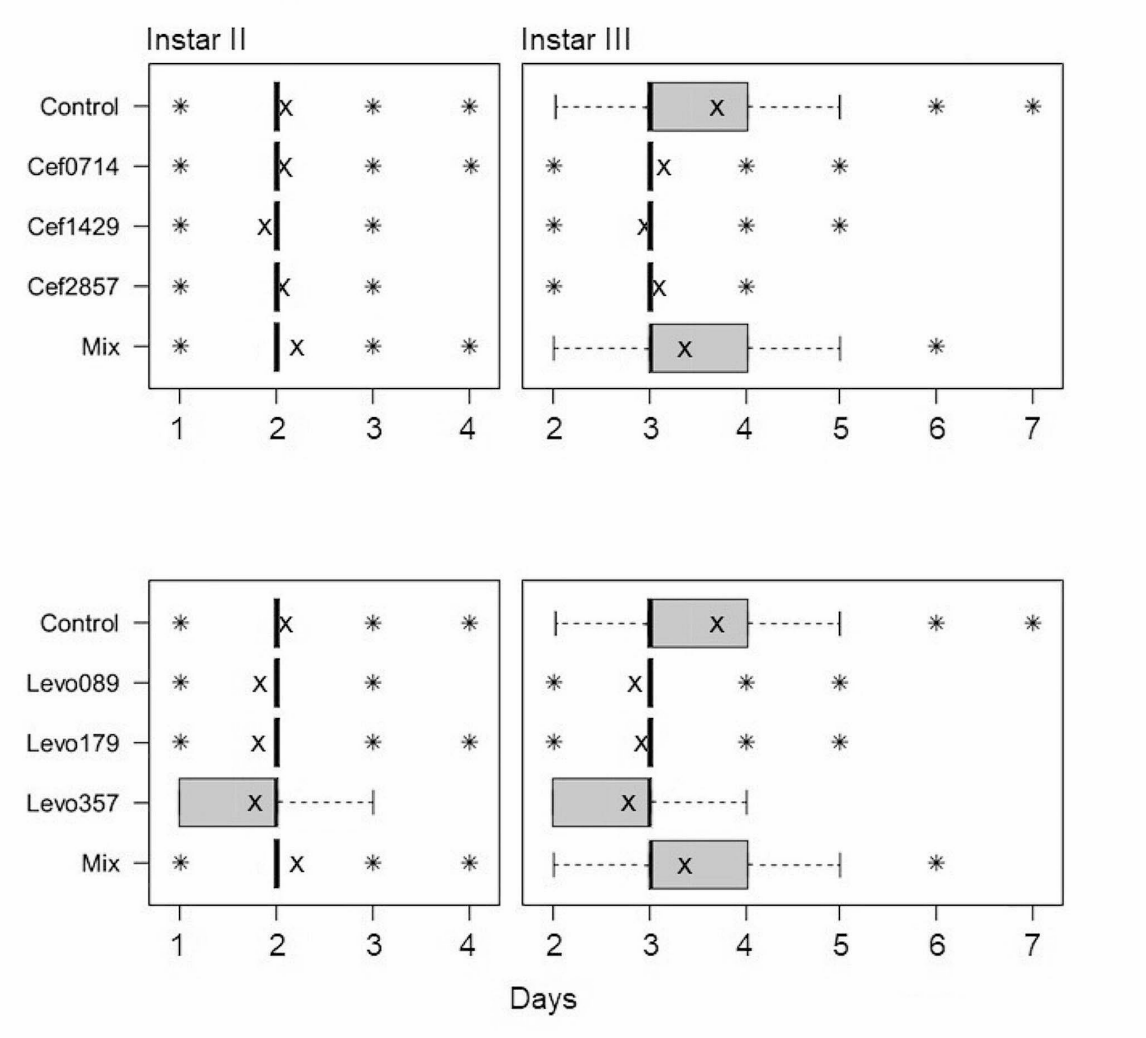
The median time required for the transition to the second and third instar, “x” marks the average time required and “*” marks outliers

The time to reach the second and third instar and the results of the statistical evaluation are shown in Fig. 5; Table 2, respectively. The transition to the second instar was significantly delayed in the control compared to every levofloxacin treatment and to the Cef 14.29 treatment. The transition to the second instar was significantly delayed in the mixed treatment compared to every other treatment. The transition to the second instar was significantly delayed in the Cef 14.29 treatment compared to the Cef 28.57 treatment. No significant differences were found between the levofloxacin treatments.

**Table 2:** *P*-values of the comparisons to reach the second (left) and third (right) instar, bold figures indicate significant differences

	Cef0714	Cef1429	Cef2857	Mix
Control	0.657 / **0.001**	**0.002** / **0.001**	0.425 / **0.001**	**0.012** / **0.001**
Cef0174		0.051 / 0.093	0.895 / 0.556	**0.018** / **0.009**
Cef1429			**0.019** / 0.139	**0.001** / **0.001**
Cef2857				**0.001** / **0.001**
	Levo089	Levo179	Levo357	Mix
Control	**0.001** / **0.001**	**0.001** / **0.001**	**0.001** / **0.001**	**0.012** / **0.001**
Levo089		0.918 / 0.223	0.484 / 0.181	**0.001** / **0.001**
Levo179			0.557 / **0.018**	**0.001** / **0.001**
Levo357				**0.001** / **0.001**

The time to reach the third instar was significantly delayed in the control compared to every other treatment. The transition to the third instar was significantly delayed in the mixed treatment compared to the levofloxacin and ceftriaxone treatments. No significant differences were found between the ceftriaxone treatments. The time to reach the third instar was significantly reduced in the Levo 3.57 treatment compared to the Levo 1.79 treatment.

### Maggot survival

**Fig. 6:**
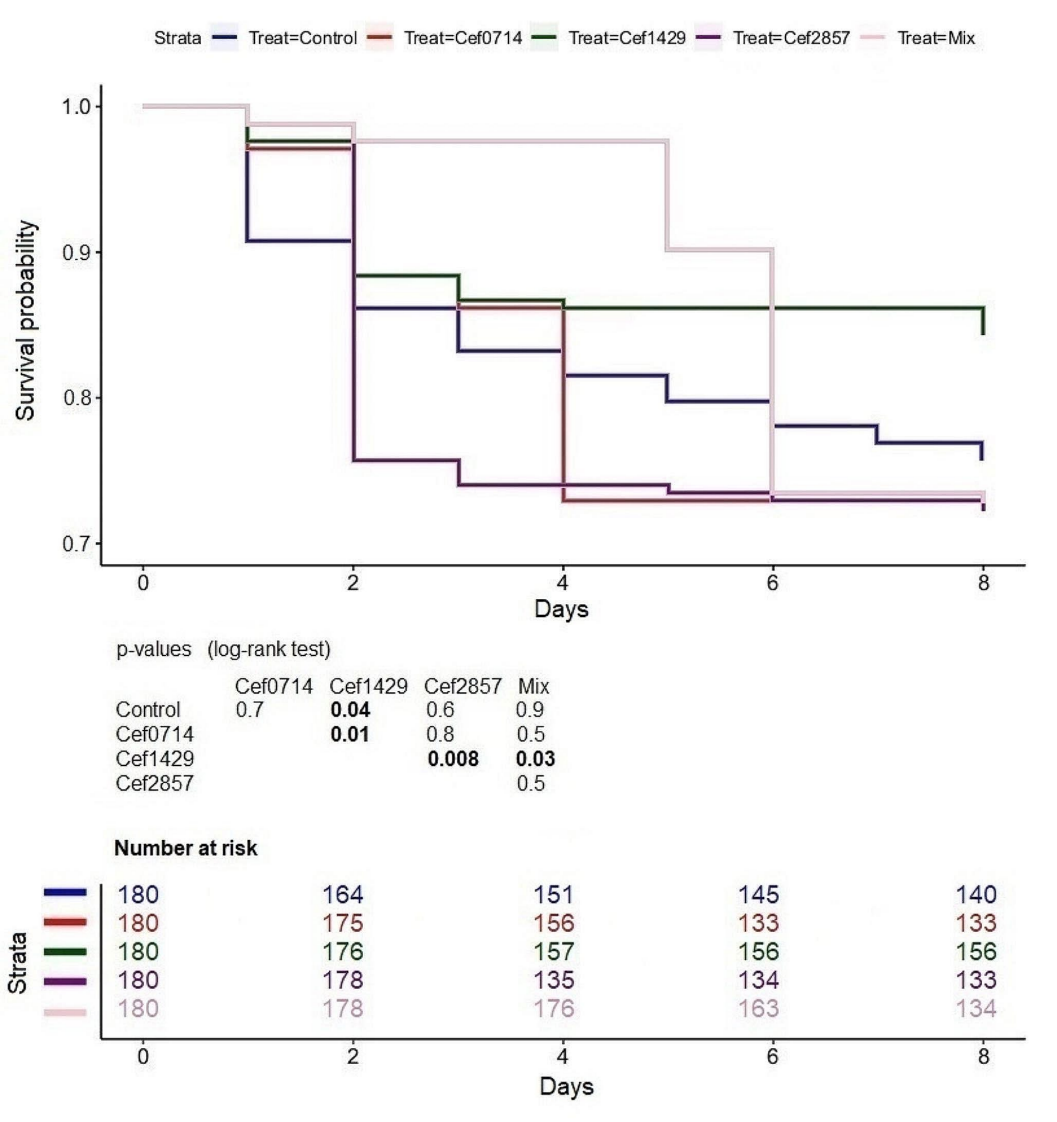
Maggot survivability (ceftriaxone), bold numbers indicate significant differences. The number of risk shows the total number of examined maggots, including the death maggots

**Fig. 7:**
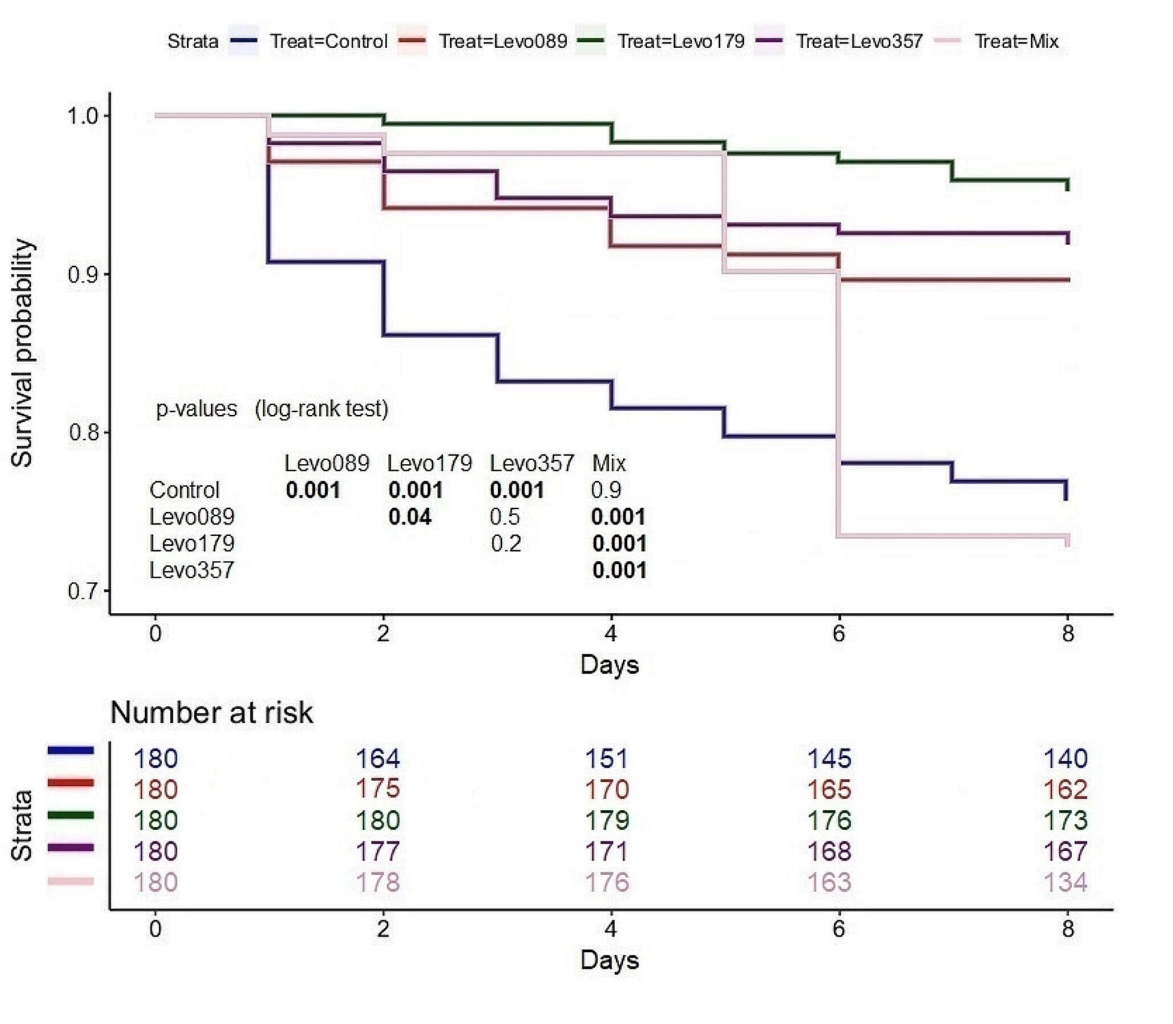
Maggot survivability (levofloxacin), bold numbers indicate significant differences. The number of risk shows the total number of examined maggots, including the death maggots

The highest mortality rate was determined in the Cef 28.57 treatment with 48 dead maggots. The second highest mortality rate was determined in the mixed treatment and the Cef 7.14 treatment with 47 dead maggots respectively (Fig. 6). The lowest mortality rate was determined in the Levo 1.79 treatment with 8 dead maggots (Fig. 7). The mortality rate in the levofloxacin treatments was significantly lower compared to the control and the mixed treatment. A significant difference was found between the Levo 1.79 and Levo 0.89 treatment. The mortality rate in the Cef 14.29 treatment was significantly lower compared to the control, the mixed treatment, and the other ceftriaxone treatments.

### Pupation

**Fig. 8:**
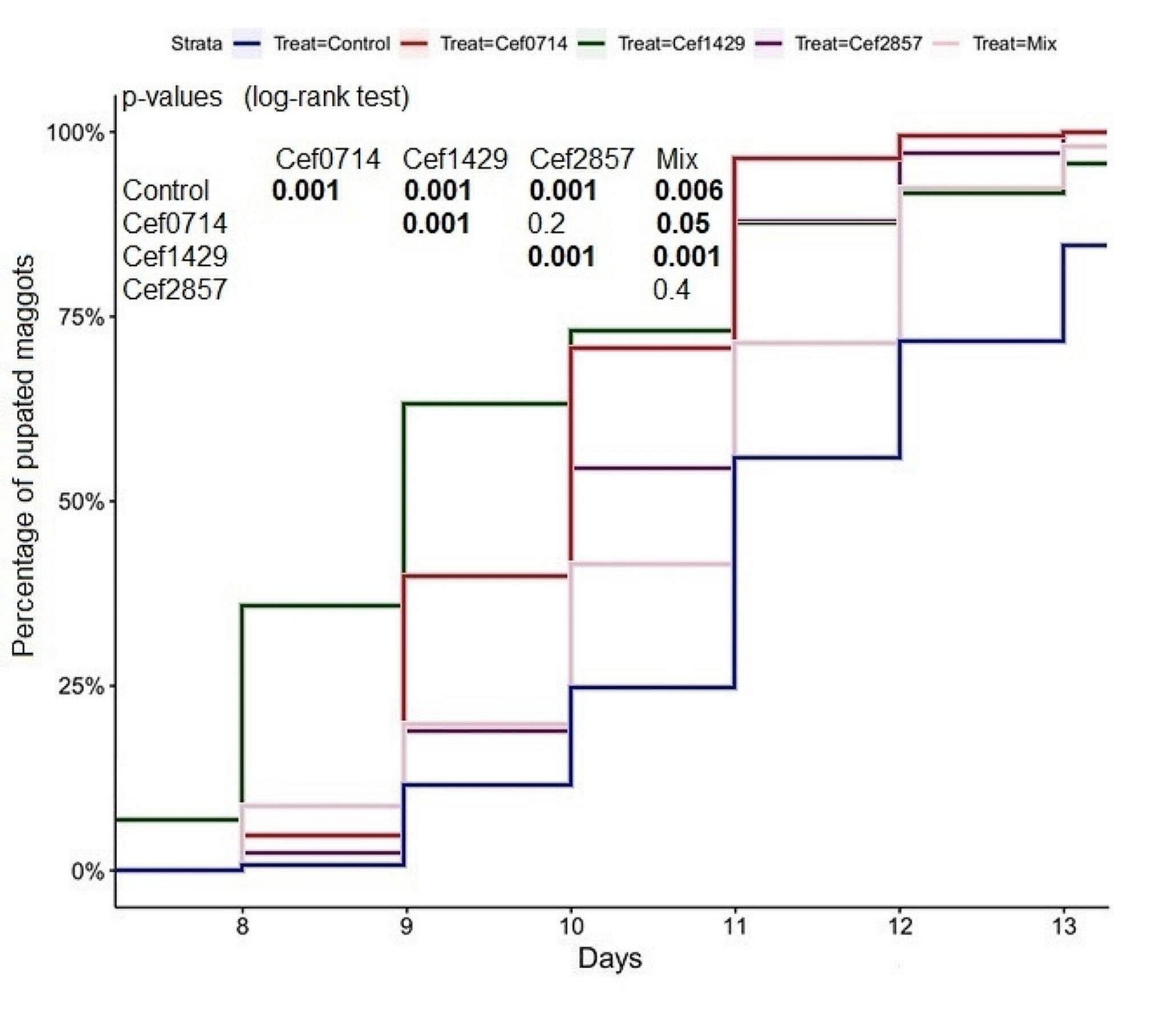
Pupated maggots (ceftriaxone), bold numbers indicate significant differences

**Fig. 9:**
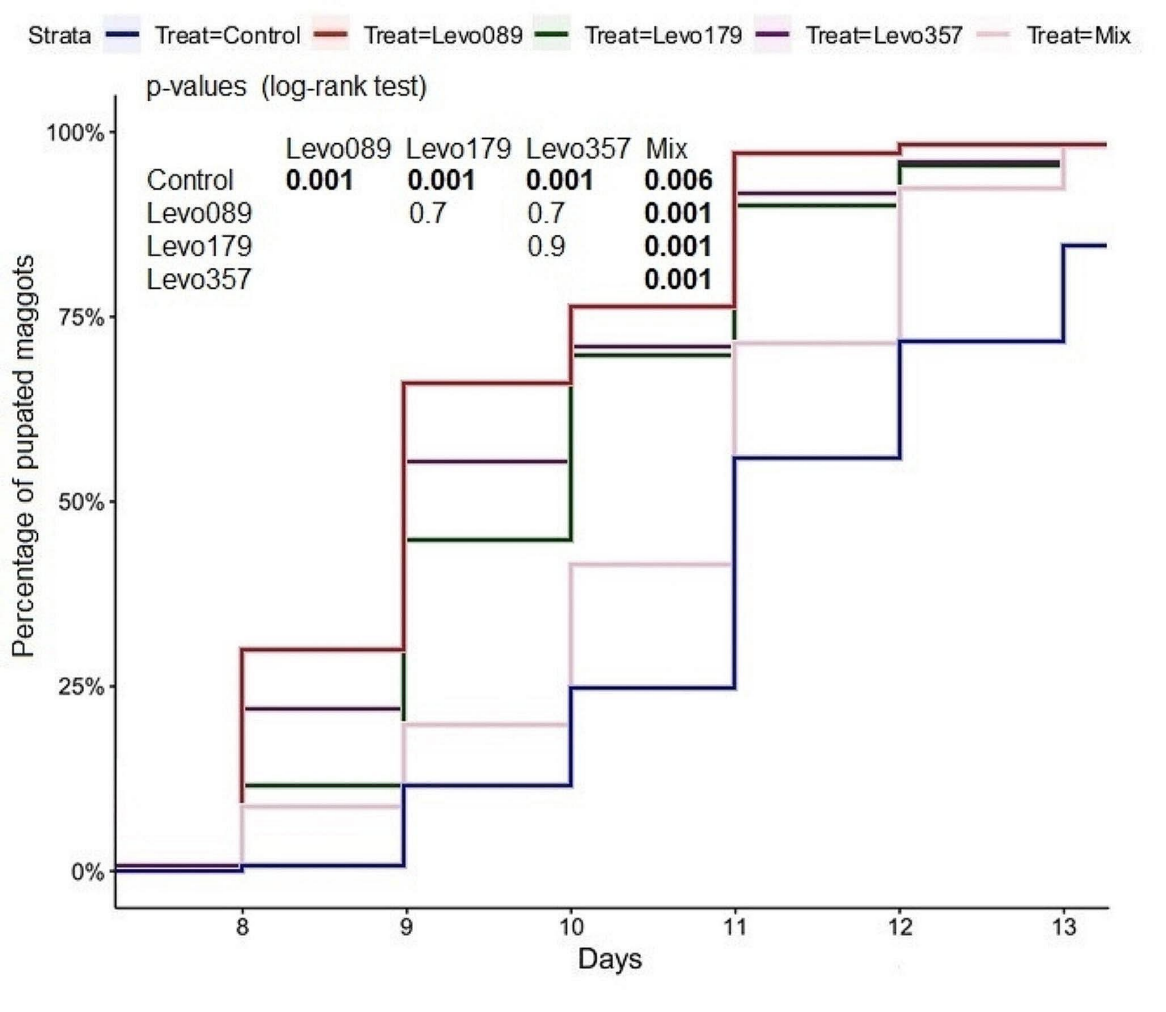
Pupated maggots (levofloxacin), bold numbers indicate significant differences

At the seventh day, the first maggots started the pupate. A significant delay of the pupation was determined in the control compared to the other treatments. From the seventh to the eleventh day, the highest pupation rate was determined in the Cef 14.29 treatment, after the eleventh day, the highest pupation rate was determined in the Cef 7.14 treatment. The pupation time of maggots in the mixed treatment was significantly delayed compared to the Cef 7.14 and Cef 14.29 treatments, and significantly increased compared to the control. No direct correlation between the pupation rate and the ceftriaxone concentration was found (Fig. 8). The pupation time of the mixed treatment was significantly delayed compared to all levofloxacin treatments. The highest pupation rate was determined in the Levo 0.89 treatment, and the second highest pupation rate was determined in the Levo 3.57 treatment. No significant differences between the single levofloxacin treatments were found (Fig. 9).

## Discussion

The antibiotics levofloxacin and ceftriaxone significantly influenced the maggot weight, length, development time and mortality. From the second day, the maggot weight and length remained significantly lower in the control compared to the levofloxacin and ceftriaxone treatments. These differences are also reflected in the development time, as the transition to the second instar was significantly delayed in the control compared to the levofloxacin treatments and the Cef 14.29 treatment. The transition to the second instar was significantly delayed in the mixed treatment compared to the control. The transition to the third instar was significantly delayed in the control compared to the mixed treatment. On the second day, maggot length and weight in the control were higher compared to the mixed treatment. From the third day, maggot length and weight in the mixed treatment were higher compared to the control. The combination of both antibiotics caused therefore a delay of the development during the first 48 h. After reaching the second instar, the development time, average weight, and length increases significantly in the mixed treatment compared to the control. The average time to reach the third instar in the control was 3.7 days, whereas the average time to reach the third instar in the Levo 3.57 treatment was 2.7 days, meaning an average difference of 24 h. As the treatment with the highest dose of levofloxacin caused the most significant reduction of the development time, it could be assumed that higher concentrations might increase the effect. However, the time for the transition to the third instar was slightly reduced in the Levo 0.89 treatment compared to the Levo 1.79 treatment. The delay of the development is also evident during pupation. From the eighth day, the total amount of pupated maggots in the Cef 14.29 treatment and the Levo 0.89 treatment is higher compared to the total amount of pupae in the control 48 h later. From the eighth day, the total amount of pupae in the treatments Levo 1.79 and Levo 3.57 is equal or higher than the total amount of pupae in the control 24 h later. The transition to the second and third instar as well as the pupation was faster in all three levofloxacin treatments compared to the control.

The time to reach the second and third instar was reduced in the Cef 14.29 treatment compared to the other ceftriaxone treatments. Until the eleventh day, a significantly reduced time to start pupation was determined in the Cef 14.29 treatment compared to the treatments Cef 7.14 and Cef 28.57. A direct correlation of the ceftriaxone concentration and the development time of the maggots cannot be verified based on our data. Similar to the levofloxacin treatments, the transition to the third instar as well as the pupation was significantly faster in all ceftriaxone treatments compared to the control.

The highest survivability was determined in the levofloxacin treatments as in all three treatments the mortality was lower or equal to 10% of the total maggot population. Based on our results, a direct correlation between the antibiotic concentration and the survivability cannot be verified as the highest survivability was determined in the Levo 1.79 treatment. Levofloxacin significantly improved the rearing conditions as it reduced the development time, increased weight and length of the maggots and increased the survivability in all levofloxacin treatments compared to the control and the mixed treatment. Only the Cef 14.29 treatment significantly increased the maggot survivability. The Cef 7.14 and Cef 28.57 treatments showed no significant difference to the control. 42 Maggots died in the Cef 28.57 treatment during the first 48 h, meaning in the first instar or during the transition to the second instar. From the second to the eighth day, only 6 maggots died in the Cef 28.57 treatment. A study on the antibacterial efficiency of *Lucilia sericata* maggots concluded that *Pseudomonas aeruginosa* is toxic to maggots and that maggot therapy was therefore ineffective in the treatment of *Pseudomonas aeruginosa* infections [21]. We suspect a similar effect in our study, as the suppression of potential pathogenic gram-negative bacteria by levofloxacin significantly improved both the survival rate and maggot development. The broader activity spectrum of levofloxacin may explain why the effects of levofloxacin were more evident than those of ceftriaxone.

Due to the forensic importance, various entomotoxicological investigations were carried out with *Protophormia terraenovae*. The effects of different temperatures were examined in various studies [3, 12, 32]. Entomotoxicological research of *Protophormia terraenovae* however is scarce. An examination of liver spiked with methylparaben revealed that the maggot development is delayed, the size of the pupae is decreased and the pupal mortality is increased by 9% [14]. The influence of ceftriaxone and levofloxacin on the Calliphoridae species *Lucilia sericata* and *Calliphora vomitoria* was examined by Preußer et al. [15, 16]. The studies concluded that therapeutical doses of levofloxacin cause an inhibition of the maggot growth of *Calliphora vomitoria* as well as a delay of the pupation. The mixed treatment amplified the delay and ceftriaxone did not significantly influence the maggot development. Both antibiotics increased the survivability of *Calliphora vomitoria* [15]. Only high concentrations of levofloxacin caused a delay of the pupation time of *Lucilia sericata* and all antibiotic treatments caused an increase of the mortality [16]. Although all three species belong to the family Calliphoridae, the effects of the used antibiotics differ significantly. The maggot length and weight of *Calliphora vomitoria* was significantly decreased by the mixture of both antibiotics, whereas the length and weight of *Lucilia sericata* remained unaltered compared to the control. However, the antibiotics caused and significant increase of the length and weight of *Protophormia terraenovae*. The required time to reach the third instar of *Protophormia terraenovae* was reduced by an average of 24 h by levofloxacin, whereas the transition to the third instar of *Calliphora vomitoria* was delayed by levofloxacin by 10 h. Different effects of the rearing conditions were also examined in the survivability as single antibiotic treatments improved the survivability of *Protophormia terraenovae* and *Calliphora vomitoria* but decreased the survivability of *Lucilia sericata* [15, 16]. Due to the secretion with the urine, levofloxacin can be accumulated near excretory organs [33]. As *Protophormia terraenovae* cause pre-mortem myiasis [8, 9], maggots could ingest levofloxacin from the tissue around the contaminated areas and their growth would be affected. As the development time is significantly reduced, levofloxacin could therefore cause an overestimation of the post-mortem interval or the time of neglect.

In addition to the forensic use, the medical benefit of maggot therapy is also increasingly researched [11, 34]. Although *Lucilia sericata* is the most commonly used species, maggot therapy was ineffective against infections caused by *Pseudomonas aeruginosa* as the bacterium has been shown to be toxic to *Lucilia sericata* maggots [21]. Tests with *Protophormia terraenovae* caused infections in patients, but these infections were subsequently traced back to a contamination of the eggs [11]. Studies show that maggot therapy is most efficient against gram-positive bacteria such as *Staphylococcus aureus*, but less efficient against gram-negative bacteria such as *Pseudomonas aeruginosa* [21]. As ceftriaxone is reported to be more effective against gram-negative bacteria including *Pseudomonas aeruginosa*, and levofloxacin providing a broad spectrum of activity against gram-positive and gram-negative bacteria [26, 35–37], a combined treatment of sterile maggots and antibiotics might improve the healing process as maggots could provide a treatment against multi-resistant bacteria and antibiotics could suppress the growth of pathogenic bacteria which are toxic to the maggots.

## Conclusions

We concluded that, the rearing conditions of *Protophormia terraenovae* are significantly improved. Especially levofloxacin increased the maggot size, reduced the development time and increased the survivability. Since both antibiotics had different effects on the maggot development of *Protophormia terraenovae* and as the effects of identical antibiotic treatments vary on distinct species of the same family, no general conclusions can be drawn about the impact of antibiotics on other species based on this data. When determining the time of oviposition and therefore either the time of death or the time of neglect, levofloxacin significantly influences the maggot development. The 24-hour reduced time to reach the third instar and 48 h reduced time to start pupation could lead to an overestimation of the time of oviposition when levofloxacin is ingested. Furthermore, the improved survival conditions of *Protophormia terraenovae* maggots may be a potential indication of medical utilization.

### Key points

1. Maggot length and weight are significantly increased by therapeutical doses of ceftriaxone and levofloxacin.
2. Levofloxacin significantly decreases the maggot development time.
3. The time required to start pupation is significantly reduced by both antibiotics.
4. Levofloxacin significantly increases the survival rate of the maggots.
